# Drugs Targeting Tumor-Initiating Cells Prolong Survival in a Post-Surgery, Post-Chemotherapy Ovarian Cancer Relapse Model

**DOI:** 10.3390/cancers12061645

**Published:** 2020-06-21

**Authors:** Brittney S. Harrington, Michelle K. Ozaki, Michael W. Caminear, Lidia F. Hernandez, Elizabeth Jordan, Nicholas J. Kalinowski, Ian S. Goldlust, Rajarshi Guha, Marc Ferrer, Craig Thomas, Jyoti Shetty, Bao Tran, Nathan Wong, Carrie D. House, Christina M. Annunziata

**Affiliations:** 1 Women’s Malignancies Branch, National Cancer Institute, National Institutes of Health, Bethesda, MD 20892, USA; brittney.harrington@nih.gov (B.S.H.); mozaki618@gmail.com (M.K.O.); michael.caminear@nih.gov (M.W.C.); hernandli@mail.nih.gov (L.F.H.); ejord006@gmail.com (E.J.); nick.kalinowski22@gmail.com (N.J.K.); cdhouse@sdsu.edu (C.D.H.); 2 The National Center for Advancing Translational Sciences, National Institutes of Health, Bethesda, MD 20892, USA; igoldlust@gmail.com (I.S.G.); rajarshi_guha@vrtx.com (R.G.); ferrerm@mail.nih.gov (M.F.); craigt@mail.nih.gov (C.T.); 3 CCR Sequencing Facility, Leidos Biomedical Research, Inc., FNLCR, Frederick, MD 21701, USA; jyoti.shetty@nih.gov (J.S.); tranb2@mail.nih.gov (B.T.); 4 CCR Collaborative Bioinformatics Resource, National Cancer Institute, National Institutes of Health, Bethesda, MD 20892, USA; nathan.wong@nih.gov; 5 Advanced Biomedical Computational Science, Frederick National Laboratory for Cancer Research, Frederick, MD 21701, USA

**Keywords:** ovarian cancer, tumor-initiating cells, ALDH, HRD, oxidative stress, maintenance therapy, relapse prevention, recurrence model

## Abstract

Disease recurrence is the major cause of morbidity and mortality of ovarian cancer (OC). In terms of maintenance therapies after platinum-based chemotherapy, PARP inhibitors significantly improve the overall survival of patients with BRCA mutations but is of little benefit to patients without homologous recombination deficiency (HRD). The stem-like tumor-initiating cell (TIC) population within OC tumors are thought to contribute to disease recurrence and chemoresistance. Therefore, there is a need to identify drugs that target TICs to prevent relapse in OC without HRD. RNA sequencing analysis of OC cells grown in TIC conditions revealed a strong enrichment of genes involved in drug metabolism, oxidative phosphorylation and reactive oxygen species (ROS) pathways. Concurrently, a high-throughput drug screen identified drugs that showed efficacy against OC cells grown as TICs compared to adherent cells. Four drugs were chosen that affected drug metabolism and ROS response: disulfiram, bardoxolone methyl, elesclomol and salinomycin. The drugs were tested in vitro for effects on viability, sphere formation and markers of stemness CD133 and ALDH in TICs compared to adherent cells. The compounds promoted ROS accumulation and oxidative stress and disulfiram, elesclomol and salinomycin increased cell death following carboplatin treatment compared to carboplatin alone. Disulfiram and salinomycin were effective in a post-surgery, post-chemotherapy OC relapse model in vivo, demonstrating that enhancing oxidative stress in TICs can prevent OC recurrence.

## 1. Introduction

Ovarian cancer (OC) is the most lethal gynecological malignancy in the United States, resulting in over 13,000 deaths annually [1]. The standard of care for OC is a combination of maximal cytoreductive surgery and platinum-based chemotherapy to which most patients initially show response, but over 70% of women with advanced stage OCs relapse [2,3,4]. In the treatment of recurrent OC, patients with platinum-sensitive tumors can receive maintenance therapies including bevacizumab (angiogenesis inhibitor) or poly (ADP-ribose) polymerase (PARP) inhibitors [5,6]. The use of PARP inhibitors has been demonstrated to significantly improve the overall survival of patients with BRCA mutations but is of little benefit to patients without homologous recombination deficiency (HRD) [5]. Maintenance bevacizumab can be used in this population, where subset analyses of GOG-218 and ICON7 clinical trials suggested an opportunity to prolong overall survival in women with ovarian cancer at high risk of recurrence (stage IV, inoperable stage III, suboptimally cytoreduced) [7,8]. Of the most commonly presented histotype of OC, high-grade serous ovarian cancer, approximately 50% of tumors have HRD and 20% of this HRD group harbor a BRCA mutation [3,9,10,11]. In platinum resistant or refractory OC there are a limited number of non-platinum second-line treatments available, most commonly taxanes, gemcitabine, anthracyclines and topoisomerase inhibitors [3]. Therefore, it would be beneficial to identify drugs that prevent relapse in women without BRCA mutations or HRD OC.

Disease recurrence is the major cause of morbidity and mortality of OC and is considered to be driven by the survival of chemoresistant, stem-like tumor-initiating cells (TICs) [12,13,14,15]. OC TICs have been defined by the expression of cell surface markers CD44, CD133, CD117 and CD24 [16,17] and high aldehyde dehydrogenase (ALDH) activity [14,17,18]. Expression of these markers has been associated with a poorer prognosis in OC [15,18,19]. Due to their elevated expression and activity in TICs, ALDHs have been investigated as a potential target in a number of cancers including those of breast, colon and lung [20,21,22,23]. ALDH activity can promote drug metabolism and efflux, contributing to chemoresistance [15,24] and therefore represents an attractive therapeutic target to prevent disease recurrence.

The metabolic plasticity of TICs and their ability to combat oxidative stress contributes to their persistence after therapy. It has been shown that OC cells can adjust their metabolic profile in response during times of cellular stress, switching between oxidative phosphorylation and glycolysis [25,26]. OC TICs may prefer oxidative phosphorylation rather than glycolysis in cellular stress in order to maintain stemness [27]. The OC tumor microenvironment also contributes to the growth of resilient TICs that adapt to oxidative stress and excessive reactive oxygen species (ROS) generated by de-adhesion, chemotherapeutic agents and hypoxia [28,29]. OC TICs may also have enhanced drug metabolism [15], anoikis resistance [29] and metabolic plasticity [30]. In environments of elevated ROS and oxidative stress, enhanced antioxidant and drug metabolism responses provide a survival advantage for TICs, but also point to a potential vulnerability [27].

Here, we employed RNA sequencing and a drug screen to investigate the differences in signaling pathways active in HR proficient, BRCA wild-type OC cell lines grown adherently or under TIC-enriching conditions and to identify potential targets unique to TICs in order to prevent disease relapse in OC. The screens indicated that OC cells grown as TICs enriched genes involved in drug metabolism and oxidative stress, and compounds that targeted these pathways showed a greater efficacy against cells grown under TIC-enriching conditions than to adherent OC cells. After primary carboplatin treatment, the compounds enhanced killing of TICs in models of relapse in vitro and in vivo, making them viable maintenance drug candidates for patients with OC.

## 2. Results

### 2.1. OC Cells Grown in Adherent or TIC-Enriching Conditions Have Different Drug Sensitivities and Pathway Activation

**Figure 1:**
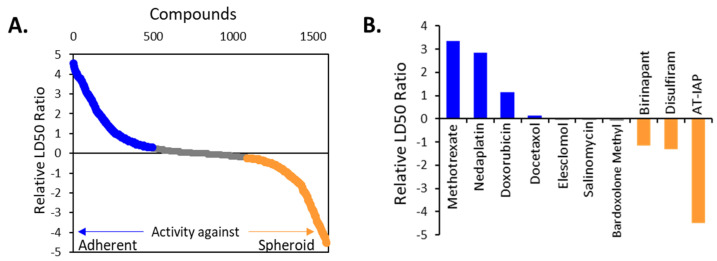

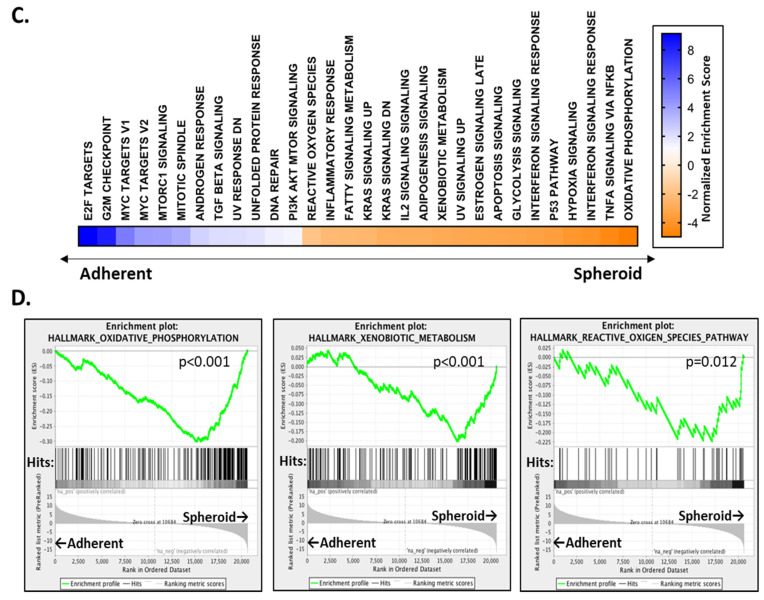
RNAseq reveals enrichment of genes in spheroids are related to oxidative stress management (**A**) Relative ratio of in vitro LD50 values for 1978 drugs in OV90 cells grown in spheroid conditions compared to those grown in adherent conditions. Orange indicates agents that were more effective on spheroids, grey represents agents that were equally effective against spheroids and adherent cells, and blue represents agents more effective against adherently grown cells. (**B**) Selected agents from B relative in vitro LAD50. (**C**) Heatmap of selected GSEA Hallmark pathways enriched in OV90 spheroids from DEG of OV90 spheroids versus OV90 Adherent cells. The color scale of the heatmap represents the ranking of the indicated pathways based on their normalized enrichment score. Orange indicates pathways enriched in spheroids and blue represents pathways enriched in adherently grown cells. (**D**) Enrichment plots of select GSEA pathways enriched in spheroids compared to adherent cells, oxidative phosphorylation, xenobiotic metabolism and reactive oxygen species pathway. The plot represents the correlations of the adherent and spheroid samples with the gene hits in the indicated pathway.

In this study we sought to identify vulnerabilities acquired by cells grown in TIC-enriching conditions compared to adherently grown cells. To do this, we first examined drug sensitivity in a high-throughput screen of 1978 compounds for cytotoxicity against OV90 cells grown adherently or in TIC-enriching sphere conditions. We have previously demonstrated that this culture technique enriches for TICs [14,17], and this cell line has been reported as HR proficient, BRCA wild-type [31]. The relative in vitro LD_50_ was calculated for each of the compounds for adherent and TIC-enriching conditions (Table S1). The log LD_50_ of each drug for adherent conditions was subtracted from the log LD_50_ of the drug for spheroid conditions to demonstrate the relative effectiveness of each drug in the two different conditions. Drugs that had a lower LD_50_ in spheroids compared to adherent conditions are represented by negative log values and those with a lower LD_50_ in adherent conditions compared to spheroids are represented by positive values (Figure 1A). Interestingly, the ALDH inhibitor Disulfiram was more than 10-fold more effective at killing TIC-enriched cells compared to adherent, and the common chemotherapy drug doxorubicin was nearly 10-fold more effective in the adherent cultures. Highlighted is a selection of drugs with differences in activity between spheroids and adherent OV90 cells (Figure 1B).

In a complementary approach, RNA sequencing was performed on OV90 cells grown in adherent culture and in TIC-enriching spheroid culture conditions. From this analysis, 6487 genes were identified as being differentially expressed using a false discovery rate (FDR) cut off of < 0.05 and fold change of > 1.5. Gene set enrichment analysis (GSEA) revealed coordinated expression of proliferative, cell cycle and checkpoint pathways in the adherently grown cells and an enrichment of metabolic and inflammatory pathways in spheroids (Figure 1C). This suggests that cells grown under these TIC-enriching conditions are metabolically active and likely generate large amounts of ROS, requiring the upregulation of genes related to the management of oxidative stress and drug metabolism. Integrated analysis of drug screen and gene expression differences in OV90 cells showed that the TICs upregulated oxidative phosphorylation genes more than glycolysis related genes (Figure 1D). These findings are consistent with recent work demonstrating that OC cancer stem cells have enhanced metabolic plasticity that allows them to survive glucose deprivation but still utilize oxidative phosphorylation [27] but this also a potential therapeutic opportunity for specific targeting of TICs [32]. Furthermore, pathways associated with ROS and xenobiotic metabolism—which includes ALDH isoenzymes—were also upregulated in TICs, suggesting that they have an adapted drug efflux and oxidative stress response to balance the demands of oxidative phosphorylation metabolism (Figure 1D).

From these results, the drugs selected for further investigation from the screen were salinomycin, elesclomol, bardoxolone methyl and disulfiram. These drugs were selected for several reasons. First, we were interested primarily in drugs effective against TICs, and this was seen with disulfiram. It was identified in the screen showing activity against spheroids more than adherent cells and while it has been classically used in the treatment of alcoholism [33].

Disulfiram inhibits ALDH, a marker of TIC populations in multiple cancers [21]. Secondly, we were interested in drugs that may show equal effectiveness against the TIC and non-TIC cell types to potentially eliminate both populations from tumors. These were bardoxolone methyl, elesclomol and salinomycin. Bardoxolone methyl has been investigated for use in the treatment of chronic kidney disease and as an inhibitor of NF-κB signaling and anti-inflammatory drug in cancer models [34]. Elesclomol has been reported to induce apoptosis in cancer cells by generating intracellular ROS, and salinomycin has been previously shown to inhibit cancer stem cells in other cancer types including breast and colon [35,36]. Therefore we investigated these drugs for their activity against OC.

### 2.2. Candidate Drugs’ Cytotoxicity against Ovarian Cancer Cell Lines was Validated In Vitro

**Figure 2:**
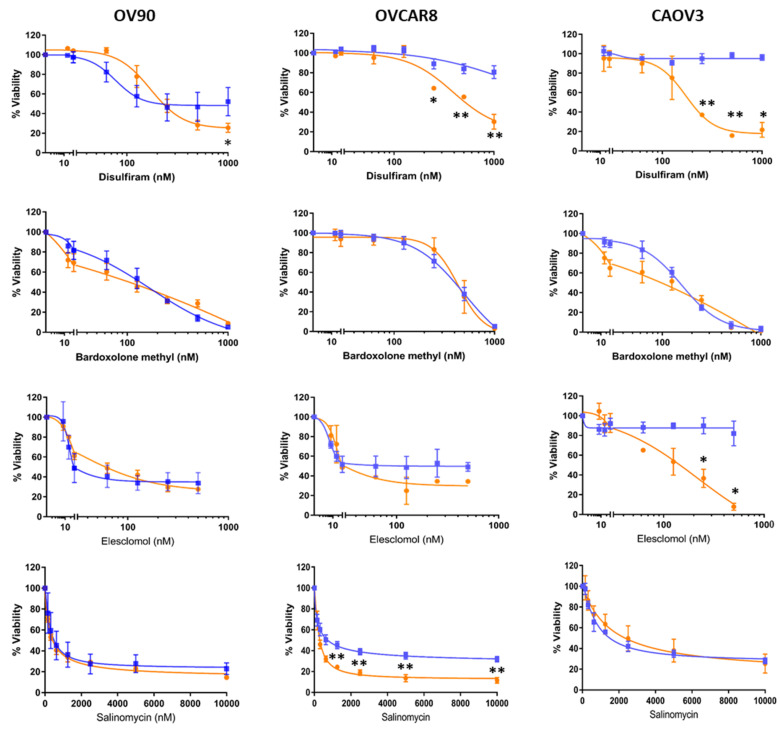
Confirmation of the candidate drug’s activity against ovarian cancer cells. Dose response curves of selected agents on additional cell lines grown in adherent (blue) or TIC-enriching spheroid (orange) culture conditions. Graphs represent mean and SEM compared to control * *p* < 0.05, ** *p* < 0.01.

The ability of disulfiram, bardoxolone methyl, elesclomol and salinomycin to affect cell viability was validated in OV90 and two other OC cell lines, OVCAR8 and CAOV3, representing platinum-resistant, HR-proficient OC [31,37]. Despite ~10% BRCA1 CpG sites showing methylation in OVCAR8, this cell line is also resistant to PARP inhibitors [38,39,40]. The viability of adherent and TIC cultured OC cells was measured after 72 h of exposure to each of the drugs in a dilution series (Figure 2). 

Disulfiram showed the most dramatic effect on cell viability of cells grown in TIC-enriching spheroid conditions compared to adherently grown cells and was consistent between the OVCAR8 and CAOV3 cell lines at a range of doses. Bardoxolone methyl was not more effective against TICs than adherent cells which is consistent with the relative LD50 ratio result obtained in the drug screen. Elesclomol showed better efficacy against CAOV3 TICs than adherently grown cells but did not demonstrate the same difference in efficacy against other cell lines grown as spheroids. Finally, salinomycin showed a small difference in efficacy against OV90 and OVCAR8 TICs compared to adherent cells, but not in CAOV3. Appreciable differences in efficacy of drugs in different cell line growth conditions were found that may be useful to target multiple cell populations (Table 1).

### 2.3. Candidate Drugs Reduced Sphere Formation

**Figure 3:**
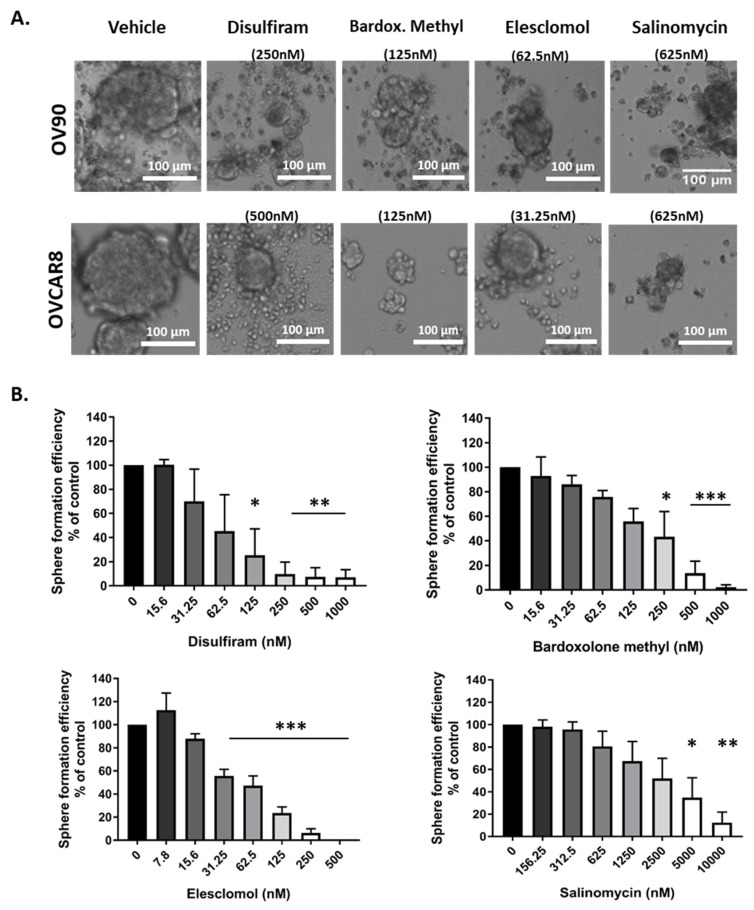
Sphere formation efficiency of ovarian cancer cells exposed to the drugs. (**A**) The drugs break apart pre-formed spheroids after exposure to drugs for 72 h in OV90 cell line (top) and OVCAR8 (bottom). (**B**) OV90 sphere formation efficiency was quantified in increasing concentrations of drugs as number of spheroids over 1000 µm^2^. Graphs represent mean and SEM Graphs represent mean and SEM of each treatment, distinguished by different degrees of grayscale shading * *p* < 0.05, ** *p* < 0.01, *** *p* < 0.001 compared to vehicle control.

Sphere formation ability is considered a phenotype of TICs and assists in OC metastasis, anoikis resistance and chemoresistance [41]. Therefore, the drugs’ effects on spheroid formation in OV90 and OVCAR8 cells were examined (Figure 3A). 

In this assay, OC cells were treated with each of the drugs at a range of concentrations 48 h after they were seeded into ultra-low attachment plates in TIC-enriching culture conditions to assess formation efficiency, rather than drugs being added to pre-formed spheroids as in the previous assay. Inhibition of sphere growth was dose dependent (Figure 3B), the dose response from Figure 2 is replicated in the sphere formation assay for disulfiram, where the highest doses inhibited sphere formation significantly, but for concentrations below the LD50 the sphere formation efficiency is less inhibited. Elesclomol had significant inhibitory activity across a broad range of concentrations tested. In comparison, the efficacy of salinomycin and bardoxolone methyl against sphere formation was only evident at higher cytotoxic doses suggesting that their activity against TICs was based on affecting viability rather than sphere formation.

### 2.4. Candidate Drugs Cytotoxicity against TIC Populations was Validated In Vitro

**Figure 4:**
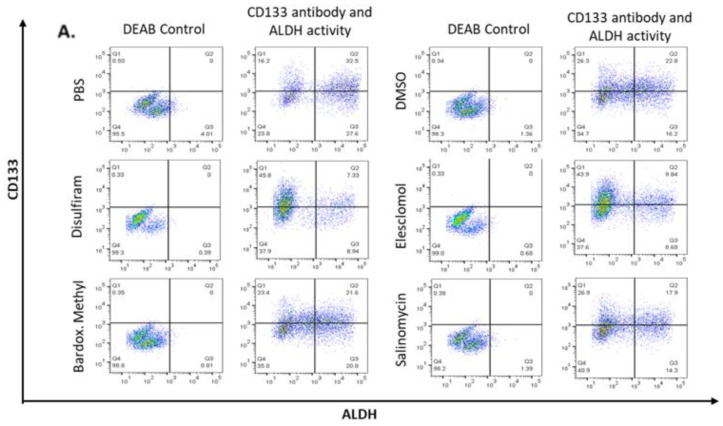

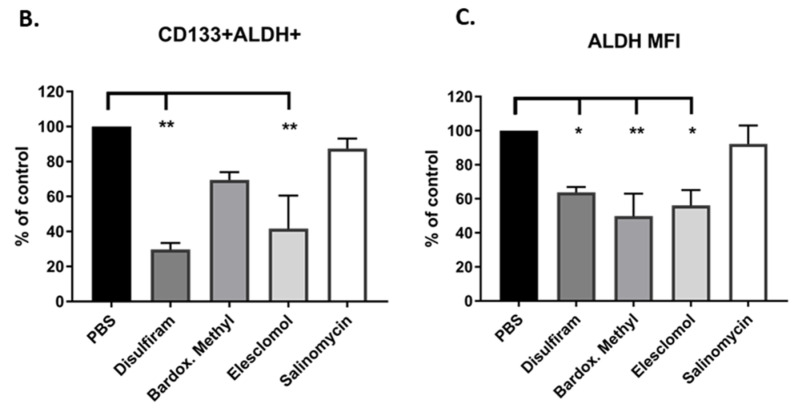
Expression of cancer stem cell markers ALDH activity and CD133 expression in OV90. (**A**) CD133 + ALDH^high^ populations of cells grown under TIC-enriching conditions after 72 h exposure to the drugs at LD50 concentrations or PBS control. (**B**) Quantified values of the CD133 + ALDH^high^ double positive population expressed as percentage of control (PBS). (**C**) Quantified geometric mean intensity of ALDH in CD133 + ALDH^high^ populations, expressed as percentage of control (PBS). Graphs represent mean and SEM of each treatment, indicated by grayscale shaded bars, * *p* < 0.05, ** *p* < 0.01.

To examine the efficacy of the drugs against TIC populations of the OC cell lines, the expression of TIC marker CD133 and high ALDH activity (CD133 + ALDH^high^) was assessed. OV90 cells were grown in TIC-enriching conditions, exposed to each drug for 48 h and then prepared for flow cytometry analysis (Figure 4A). Disulfiram and elesclomol significantly decreased the CD133 + ALDH^high^ population, suggesting these drugs were able to target the TICs within spheroids (Figure 4B). 

Interestingly, examining ALDH activity of the whole population showed bardoxolone methyl, in addition to disulfiram and elesclomol, significantly decreased ALDH activity (Figure 4C), but none of the drugs significantly affected CD133 expression in the whole population. This suggests that disulfiram and elesclomol directly suppress the TIC population and disrupt ALDH activity.

### 2.5. Candidate Drugs Enhance the Oxidative Stress of Cells Grown in TIC-Enriching Spheroid Conditions

**Figure 5:**
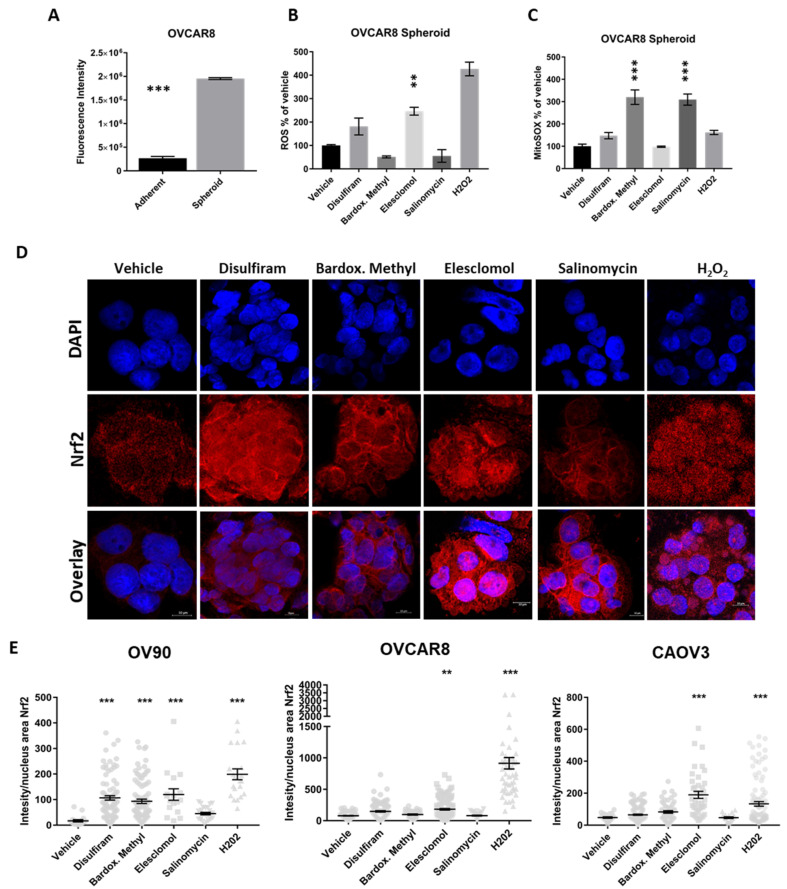
Candidate drugs cause oxidative stress in OC spheroids. (**A**) Ovcar8 cells grown in TIC-enriching spheroid conditions have significantly higher levels of ROS compared to adherent Ovcar8. (**B**) Ovcar8 spheroids treated with the drugs at LD50 for 6h showed elesclomol produced higher levels of intracellular ROS compared to vehicle. (**C**) Ovcar8 spheroids treated with LD_50_ concentrations of the drugs were analyzed for MitoSOX levels. Bardoxolone methyl and salinomycin for 6 h showed increased MitoSOX compared to control. (**D**) Representative images of Nrf2 (red) translocation to the nucleus (blue, DAPI) in OV90 spheroids after 6h treatment with each of the drugs at respective LD50 doses. 63X objective, scale bar is 10μm. (**E**) Quantification of Nrf2 nuclear localization in each cell line treated with each of the drugs for 6h. Graphs represent mean and SEM compared to control ** *p* < 0.01, *** *p* < 0.001.

Based on the pathways identified in gene expression analyses, we asked whether the candidate drugs were able to induce oxidative stress on OC cells grown as spheroids and exceed their ability to manage ROS. Intracellular ROS was first examined in OVCAR8 cells grown adherently or in TIC-enriching spheroid conditions which showed that the latter have higher baseline levels of oxidative stress compared to adherent cells (Figure 5A). Focusing on the spheroids, the effect of each of the drugs on intracellular ROS levels was examined after 6 h exposure at LD50 concentrations and was compared to vehicle control (Figure 5B). Elesclomol significantly increased intracellular ROS and this finding is consistent with prior studies on the activity of this drug [35,36]. Disulfiram appeared to increase intracellular ROS but not to a significantly higher level compared to vehicle. In contrast, salinomycin and bardoxolone methyl appeared to reduce ROS compared to vehicle in the 6 h treatment time, but after 24 h exposure to the drug intracellular ROS was increased above vehicle. Adherent cells treated with the drugs did not accumulate ROS at levels greater than background, likely because the ROS clearance in adherent cells is balanced compared to spheroids (Figure S1A) [41]. Mitochondrial superoxide production was also examined in the spheroids treated with the each of the drugs for 6 h (Figure 5C). Interestingly, bardoxolone methyl and salinomycin treatment induced mitochondrial superoxide (MitoSOX) production and was significantly higher than vehicle control suggesting that these drugs may target the mitochondria to enhance oxidative damage. None of the drugs significantly increased MitoSOX in adherent cells (Figure S1B), again suggesting spheroids are more susceptible to oxidative stress.

Nrf2 nuclear translocation is also a marker of oxidative stress. In the nucleus Nrf2 activates the antioxidant response elements to promote transcription of genes that protect cells from ROS toxicity [42]. We examined the nuclear translocation of Nrf2 in spheroids collected by cytospin and counterstained with DAPI to distinguish the nucleus (Figure 5D). Co-staining of Nrf2 and DAPI indicates nuclear localization of Nrf2 in elesclomol-, disulfiram- or bardoxolone-treated OV90 spheroids, whereas cytosolic localization of Nrf2 was observed in salinomycin and vehicle treated spheroids. Nrf2 nuclear translocation was quantified for each of the cell lines with the indicated treatments (Figure 5E). In all the cell lines, elesclomol caused the most nuclear localization of Nrf2 compared to vehicle and was comparable to hydrogen peroxide (H_2_O_2_) which was the positive control treatment. Disulfiram and bardoxolone methyl were significantly higher than control only in the OV90 spheroids, suggesting that OV90s are more sensitive to ROS and oxidative stress than the other cell lines. Salinomycin caused very little localization of Nrf2 in the nucleus in any of the cell lines. Salinomycin has been shown to inhibit Nrf2 translocation and our results indicate this is true in OC spheroids also [43].

### 2.6. Combination of Drugs with Carboplatin Decreased TIC Populations In Vitro

**Figure 6:**
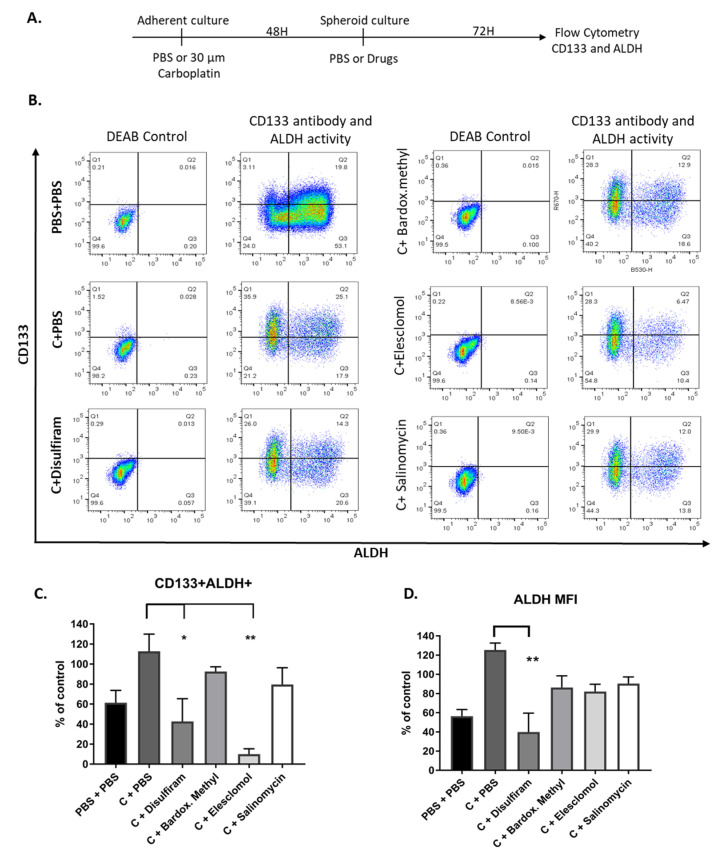
Drugs effect on CD133 + ALDH^high^ populations after carboplatin treatment. (**A**) Schematic of the assay of OV90 cells treated with 30μM carboplatin under adherent conditions for 48 h, and then switched to TIC-enriching spheroid growth conditions and treated with the candidate drugs at LD_50_ concentrations for a further 72 h. (**B**) Representative images of CD133 + ALDH^high^ populations in each treatment group. (**C**) CD133 + ALDH^high^ double positive population expressed as percentage of control (Carboplatin + PBS). (**D**) ALDH geometric mean intensity, expressed as percentage of control (Carboplatin + PBS). Graphs represent mean and SEM Graphs represent mean and SEM of each treatment, distinguished by different degrees of grayscale shading compared to control * *p* < 0.05 ** *p* < 0.01.

TICs persist after chemotherapy treatment and repopulate tumors and we have previously shown that ALDH activity increases in TIC-grown cells in response to carboplatin [14]. Therefore, we were interested to investigate whether after treatment with carboplatin, the drugs could reduce ALDH activity and CD133 expression which was measured by flow cytometry (Figure 6A,B). Following treatment with carboplatin, elesclomol and disulfiram significantly decreased the percentage of CD133+ ALDH^high^ cells and importantly, none of the drugs increased ALDH activity in combination with carboplatin (Figure 6C). 

None of the drugs in combination with carboplatin affected CD133 expression in the whole population. The ALDH activity of the whole population was significantly reduced only with disulfiram and carboplatin treatment, confirming the activity of the drug inhibiting ALDH activity was not inhibited by carboplatin (Figure 6D).

### 2.7. Drugs Targeting the TIC Population Prevent/Reduce Relapse In Vitro and In Vivo

**Figure 7:**
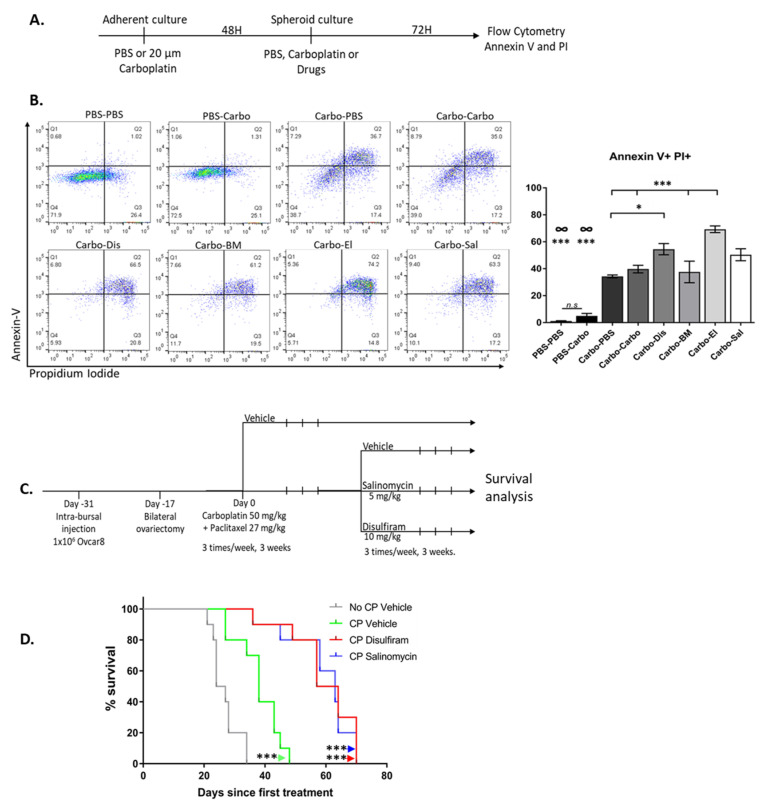
Drugs effect on models of relapse in vitro and in vivo. (**A**) Schematic of the in vitro relapse model of OVCAR8 cells treated with PBS or 20 μM carboplatin under adherent conditions for 48 h, and then switched to TIC-enriching spheroid growth conditions and treated with the candidate drugs at LD50 concentrations, carboplatin at 20 μM or PBS for a further 72 h. (**B**) Representative images (left panel) of Annexin V and PI staining that was used to quantify cell death (right panel) Carbo = Carboplatin, Dis = Disulfiram, BM = Bardoxolone Methyl, El = Elesclomol, Sal = Salinomycin. Graphs represent mean and SEM Graphs represent mean and SEM of each treatment, distinguished by different degrees of grayscale shading, n.s = not significant, ∞ = compared to all groups, * *p* < 0.05, *** *p* < 0.001. (**C**) Schematic of the in vivo relapse model. (**D**) Kaplan-Meier survival analysis of mice treated with vehicle only (grey, “No CP Vehicle”), Carboplatin and Paclitaxel (green, “CP Vehicle”), Carboplatin and Paclitaxel followed by Disulfiram (red, “CP Disulfiram”) and Carboplatin and Paclitaxel followed by Salinomycin (blue, “CP Salinomycin”). Survival was measured in days since the first treatment *** *p* < 0.001 compared to vehicle control.

To test the efficacy of the drugs against recurrence, we created an in vitro relapse model. Adherent cells were cultured with carboplatin for 48 h, then grown in TIC-enriching spheroid conditions in the presence of drugs, and cell death was measured with propidium iodide and annexin V after 3 days (Figure 7A,B). After carboplatin treatment, elesclomol significantly enhanced cell death, compared to vehicle controls or a second exposure to carboplatin. Disulfiram increased cell death in combination with carboplatin and was better than exposure to carboplatin alone with no second exposure. Salinomycin treatment increased cell death compared to PBS-treated cells but did not increase cell death above what was observed with carboplatin alone. Interestingly, treating cells with carboplatin in TIC-enriching conditions did not significantly increase cell death compared to PBS-treated controls, but carboplatin treatment before sphere-formation did increase cell death. This is consistent with the increased drug resistance of OC spheroids due to reduced drug penetrance that has been described [14,41,44].

We next optimized a mouse model of ovarian cancer in order to investigate relapse in vivo (Figure S2A). The mice were inoculated with OVCAR8 cells into the right ovarian bursa and PBS into the left ovarian bursa and allowed to recover for 2 weeks, then bilateral ovariectomies were performed. Mice recovered from surgery for two weeks and then commenced 1, 2 or 3 cycles of intraperitoneal carboplatin-paclitaxel treatment. Mice that received 1 or 2 cycles did not survive longer than vehicle treated mice (Figure S2B). Those treated with 3 cycles had significantly greater median overall survival, but all mice eventually succumbed to their disease, closely mimicking the clinical course of patients with platinum-resistant OC. We proceeded to use this model to test the ability of maintenance drugs to prevent disease recurrence in vivo. Mice underwent intra-bursal inoculation, surgical resection and chemotherapy as described above. After completing chemotherapy, mice received 3 weeks of treatment with vehicle, Disulfiram or Salinomycin, and then were monitored for survival (Figure 7C). These drugs were chosen for the in vivo model to examine whether Salinomycin, a drug that showed efficacy against both adherent and TIC viability in vitro, could provide benefit by targeting both populations in a tumor compared to Disulfiram which targeted TICs. Elesclomol was excluded from the in vivo relapse model because of unacceptable toxicity to mice when given in long-term maintenance fashion. Importantly, both Salinomycin and Disulfiram treatment following carboplatin-paclitaxel significantly prolonged survival compared to carboplatin-paclitaxel alone (Figure 7D).

This demonstrates these maintenance drugs provide a significant survival benefit in a mouse model of HR proficient, chemotherapy-treated OC relapse, for which clinically few treatment options are available.

## 3. Discussion

The majority of advanced OCs will relapse and become resistant to platinum chemotherapy, after which there are limited options to provide survival benefit for patients without BRCA mutations. In this study we sought to identify strategies for this OC patient population that could be used in the setting of first remission, with the goal of eradicating putative TICs and preventing relapse. We used OC cell lines that form spheres, have been reported to be resistant to platinum and PARP inhibitors, and do not carry BRCA mutations: OV90, OVCAR8 and CAOV3 [37,45,46]. We showed the efficacy of these drugs in models of relapse after chemotherapy treatment in vitro and in vivo for their ability to prolong survival.

We integrated results from global gene expression profiling by RNAseq and a comprehensive functional screen of almost 2000 pharmaceutical compounds and identified four candidate drugs to prevent OC relapse. Each drug showed activity against OC cells grown in TIC-enriched spheroid conditions in vitro, reflecting the state of disease remaining in the peritoneal cavity following initial treatment of OC. We investigated their effects on inhibiting TIC characteristics in terms of viability in adherent versus spheroid culture, sphere formation and expression of stem markers CD133 + ALDH^high^. We have previously shown that ALDH activity in OC TICs increased after exposure to platinum drugs, and that both ALDH1A1 and ALDH1A2 were upregulated in OC TICs [14]. Elevated ALDH expression protects drug resistant TICs from elevated levels of ROS and inhibition of ALDH can cause ROS accumulation and genotoxic stress [47]. Disulfiram was recently investigated for its inhibitory effects on ALDH+ cell populations in OC in combination with cisplatin and was similarly found to increase intracellular ROS, which was further enhanced by the addition of exogenous copper [33]. In this study we showed that disulfiram did increase ROS in vitro but importantly it significantly reduced OC relapse in vivo after carboplatin treatment. Disulfiram is approved for the clinical treatment of alcoholism and is delivered orally in that setting, however for the treatment of OC, oral delivery and absorption would likely not reach the tumor sufficiently as it would be first metabolized by the liver where ALDH1 and ALDH2 are highly expressed [48]. In this study we utilized disulfiram as a tool compound to inhibit ALDH activity and directly delivered the drug intraperitoneally to target the xenograft tumors. Further investigation of ALDH inhibition for OC treatment would require reformulation for intraperitoneal delivery to reach the tumors. Furthermore, it will be necessary to determine whether specific targeting of ALDH isoenzymes such as ALDH1A1, which has shown activity against OC cells in other studies [6,49,50], will be of superior benefit to broader inhibition by disulfiram. ALDH1A1 is overexpressed in several malignancies and TICs, which are associated with chemoresistance, poor prognosis, and tumor aggressiveness [51,52]. ALDH1A1 inhibitor analogs have been identified, developed, and optimized for potent cellular activity and appropriate pharmacokinetic properties [50]. The efficacy of these analogs in cancer cell in vitro models has been characterized and further comparison between the developed ALDH1A1 inhibitors and disulfiram is needed. In addition to the reported inhibition of ALDH, disulfiram has been shown to modulate the activity of other proteins and cellular processes such as ROS, Pgp and proteasome function, and the JNK signaling pathway [53,54,55]. Comparison of disulfiram and its broader cellular effects, to the effects of specific ALDH1A1 inhibitors will provide insight into which treatment is more suitable as a therapeutic to eradicate TICs from tumors and prevent OC relapse.

The drugs were investigated for their effects on oxidative stress by measuring intracellular ROS accumulation, Nrf2 activation and mitochondrial superoxide production. Compared to adherently grown cells, TICs have increased ROS at a basal level [27] and in TICs, elesclomol increased the level of intracellular ROS further within 6 h of treatment. Disulfiram only slightly increased the intracellular ROS levels in TICs within the 6 h treatment time but both elesclomol and disulfiram caused Nrf2 activation in TICs, whereas salinomycin blocked Nrf2 activation as has been described [43]. It has been demonstrated that there is a high level of heterogeneity across ovarian cancer cell lines with respect to the baseline metabolic profiles of chemotherapy resistant cells [25] and therefore would warrant an extensive evaluation to draw significant conclusions as to the metabolic activities of the four drugs. This can be pursued in a future study along with specific ALDH inhibitors. The findings of the drug screen identifying drugs that show better activity against TICs match the findings of the gene expression analysis presented here, and the recent findings of others, indicating that enhancing oxidative stress could be a target to eliminate TICs.

In the relapse models used to investigate the drugs’ effectiveness as maintenance therapy after treatment with carboplatin, elesclomol and disulfiram again showed the greatest increase in cell death over carboplatin alone in vitro. Carboplatin treatment was effective against OC cells grown in TIC-enriching conditions, but only when carboplatin was given to cells before they formed spheroids. It is interesting to note that the results of the relapse model reflect the flow cytometry and sphere formation results, in which elesclomol and disulfiram showed the greatest efficacy against TIC populations. However, despite the activity of elesclomol against TICs in vitro, it was excluded from the in vivo model of relapse because of its intolerability in mice. Elesclomol has similarly performed poorly in different in vivo models for other cancers, including those where subcutaneous administration was given, which does not require the same solubility for intraperitoneal or intravenous delivery [56,57]. In addition, findings from a clinical trial showed that in combination with paclitaxel, elesclomol did not provide any benefit in patients with platinum-resistant recurrent ovarian, tubal or peritoneal cancer [58], suggesting that elesclomol in its current formulation is not effective in humans and should therefore be reformulated before further testing. Disulfiram efficiently inhibited relapse in vivo as a maintenance therapy after carboplatin-paclitaxel which was consistent with what was observed in vitro. Salinomycin showed limited efficacy against the expression of TIC markers in vitro, however in vivo it demonstrated significant survival benefit compared to chemotherapy treatment alone. Salinomycin has shown efficacy against cancer stem cells and has shown some selectivity for cancer cells over normal cells but has not been investigated clinically to date [32].

In this study we have shown that drugs augmenting oxidative stress are effective against TICs and prevent relapse after carboplatin-paclitaxel chemotherapy in vivo. The investigated drugs disulfiram, bardoxolone methyl, elesclomol and salinomycin were used as molecular tools in this study with the aim to target the TIC population that is responsible for disease recurrence. Further development of compounds to enhance oxidative stress and inhibit ALDH activity with improved potency and better pharmacokinetic properties could provide new therapeutic options for maintenance therapy in the relapse setting. This is an area of great need, in particular to BRCA wild-type patients and patients that do not respond to PARP inhibitor therapy.

## 4. Materials and Methods

### 4.1. Antibodies and Reagents

Carboplatin (cat. No. 2626) was purchased from Tocris Bioscience (Minneapolis, MN, USA) and dissolved in phosphate buffered saline (PBS). Salinomycin (S6201) was purchased from Millipore Sigma (Burlington, MA, USA) dissolved in dimethylsulfoxide (DMSO). Disulfiram (3807) was purchased from R&D Systems (Minneapolis, MN, USA) and dissolved in DMSO. Elesclomol (S1052) and bardoxolone methyl (S8078) were purchased from Selleck Chemicals (Houston, TX, USA) dissolved in DMSO. Nrf2 antibody (ab206893) was from Abcam, (Cambridge, MA, USA). CD133 antibody (293C3) was from Miltenyi Biotec (Auburn, CA, USA). Propidium Iodide (PI) was from Roche (Cambridge, MA, USA) and AnnexinV-FITC (556420) was from BD Biosciences (San Jose, CA, USA).

### 4.2. Cell Lines and Culture Conditions

Ovarian cancer lines OV90, OVCAR8 and CAOV3 were obtained from the American Type Culture Collection (ATCC, Manassas, VA, USA). All cultures were maintained at 37 °C in 5% CO2. OV90, CAOV3, and OVCAR8 cells were cultured in RPMI (Thermo Fisher Scientific, Waltham, MA, USA) medium containing 10% (*v/v*) fetal calf serum (FCS), penicillin (100 units per mL) and streptomycin (100 units per mL). TIC-enriching culture conditions were defined as described [14,17] by maintaining cells in ultra-low attachment (ULA) plates or flasks (Corning, NY, USA) in TIC-enriching medium with reagents from Thermo Fisher Scientific: DMEM-F12 media supplemented with 1% KnockOut serum replacement, 1% penicillin/streptomycin, 0.1% insulin-transferrin-selenium, and 0.4% bovine serum albumin (A9418) and growth factors EGF 20 ng/mL (E9644) and FGF 10 ng/mL (F0291) from Millipore Sigma (Burlington, MA, USA). Cultures were grown for 3 days before experiments involving drug treatments were performed.

### 4.3. RNA Sequencing and Differential Gene Expression Analysis

Total RNA was isolated as described previously [14] from 4 independent cultures of OV90 cells grown adherently in 75 cm flasks in RPMI or in TIC-enriching culture conditions in 75 cm ULA flasks (3814 Corning). RNA quality was assessed on an Agilent Bioanalyzer (Agilent Technologies, Santa Clara, CA, USA) to ensure samples having high quality score (RIN > 9) were used for analysis. From this, 100 ng RNA was input to an mRNA capture with oligo-dT coated magnetic beads. The mRNA was fragmented for random-primed cDNA synthesis to generate the library after end-repair, adapter ligation and PCR amplification (Illumina library prep TruSeq Stranded mRNA LT library prep kit, Illumina, San Diego, CA, USA) [59]. The final purified product was quantitated by qPCR before cluster generation and paired-end sequencing on the HiSeq sequencer for 150bp read length, 2 × 150 cycle run at the CCR Sequencing Facility (Leidos Biomedical Research, Frederick, MD, USA). The samples had between 22 to 39 million pass filter reads with a base call quality of above 93% of bases with Q30 and above. The sequencing quality of the reads was assessed per sample using FastQC (version 0.11.5) (http://www.bioinformatics.babraham.ac.uk/projects/ fastqc/), Preseq (version 2.0.3) (https://github.com/smithlabcode/preseq) [60], Picard tools (version 1.119) (https://broadinstitute.github.io/ picard/) and RSeQC (version 2.6.4) (http://rseqc.sourceforge.net/) [61]. Reads were then trimmed using Cutadapt (version 1.14) (https://cutadapt.readthedocs.io/en/stable/) [62] prior to mapping to the hg19 human genome using STAR (version 2.5.2b) (https://github.com/alexdobin/STAR) [63] in two-pass mode. Overall expression levels were quantified using RSEM (version 1.3.0) (https://deweylab.github.io/RSEM/) [64]. limma (version 3.34.9) [65] was used for differential expression analysis. For differential gene expression, FDR < 0.05 and absolute fold change > 1.5 was used to identify significantly altered genes. Pathway analysis was performed using Gene Set Enrichment Analysis (v3.0) (https://www.gsea-msigdb.org/gsea/index.jsp) [66,67].

### 4.4. High-Throughput Drug Screen

The high-throughput drug screen was performed at the National Center for Advancing Translational Sciences (NCATS, Rockville, MD, USA) [68,69]. OV90 cells were seeded in 5 μL of either RPMI for adherent cell growth conditions into a 1536 well tissue culture treated microplate or TIC-enriching growth media into 1536 well spheroid microplate, using a Multidrop Combi dispenser. The TICs were allowed to form spheroids for 5 days before dispensing, but adherent cells were plated at a density of 500–1000 cells/well, to allow for compounds to be present during exponential growth phase. After cell addition, 23 nL of MIPE 5.0 compounds were added to individual wells via a 1536 pin-tool. Bortezomib (final concentration 2.3 µM) was used as a positive control for cell cytotoxicity. A total of 11 custom concentrations were examined to construct a complete response analysis of each compound. Plates were incubated for 48 h at standard incubator conditions, covered by a stainless steel gasketed lid to prevent evaporation. 3 μL of Cell Titer Glo (Promega, Madison, WI, USA) were added to each well and plates were incubated at room temperature for 15 min with a stainless-steel lid in place. Luminescence readings were taken using a Viewlux (PerkinElmer, Waltham, MA, USA) with a 2 s exposure time per plate. Compound dose response curves were normalized to DMSO and empty well controls on each plate.

### 4.5. Cell Viability

Cell viability was assessed using CellTiter-Glo (Promega, Madison, WI, USA) according to manufacturer’s instructions. Briefly, adherent cells were seeded at 2000 cells/well in 96-well white plates and incubated at 37 °C overnight. Indicated drugs were added the following day and cells were allowed to grow for 72 h before luminescence was measured. For viability in TIC-enriching conditions, cells were seeded in a ULA flask in TIC-enriching media for 3 days before being seeded at 2000 cells/well in 96-well ULA plates and treated with the indicated drugs and allowed to grow for 72 h. CellTiter-Glo was added to the spheroids in the ULA culture plates for 10 min at RT protected from light, before transferring the media to a white plate in which luminescence was measured. Luminescence was measured using a SpectraMax iD3 (Molecular Devices, San Jose, CA, USA) microplate reader.

### 4.6. Spheroid Formation Efficiency

OV90 and OVCAR8 cells were seeded at 2000 cells/well in 96-well ULA plates (Corning 3474). Cells were cultured in TIC-enriching conditions with indicated drugs for 7 days, fresh culture medium containing growth factors and the drugs was replenished every 48 h. After 7 days the spheres were imaged using the Celigo microplate cell cytometer (Nexcelom Biosciences, Lawrence, MA, USA) and using Fiji (ImageJ) software (https://fiji.sc/), spheroids measuring an area of >1000 μm^2^ were counted.

### 4.7. Flow Cytometry and ALDH Activity Assay

Cells were grown in adherent conditions, trypsinized, and 1.0 × 10^6^ cells were plated in ULA flasks (Corning, NY, USA) in TEM for 48 h then re-plated in fresh TIC-enriching media and treated with the indicated drugs. To assess cell death, the cells were collected and a total count was performed, and 1.0 × 10^5^ cells from each treatment were stained with Annexin V-FITC according to the manufacturer’s protocol. After the final wash step, PI was added at 1:10 dilution in PBS and incubated for 15 min, protected from light and directly after was analyzed on a flow cytometer. The ALDEFLUOR kit from Stem Cell Technologies (Seattle, WA, USA) was used to assess ALDH enzymatic activity, according to the manufacturer’s instructions and as has been previously described [14,70]. Briefly, 1.0 × 10^5^ viable cells from each treatment were incubated with ALDEFLUOR assay buffer containing the active substrate for 30 min at 37 °C or were incubated with a specific ALDH inhibitor diethylaminobenzaldehyde (DEAB) (30 μM) to serve as a negative control in tandem. Following staining for ALDH, cells were incubated with CD133 antibody and stained as previously described [14]. Fluorescence was detected on a flow cytometer, and analyzed using FlowJo 10 software (Becton, Dickinson and Company, Franklin Lakes, NJ, USA).

### 4.8. Immunofluorescence

Cells grown in TIC-enriching conditions for 3 days were treated with the indicated drugs for the indicated times and collected for immunofluorescent staining by cytospin at 800 rpm for 3 min. The slides were immediately fixed with 1% paraformaldehyde in PBS for 10 min at RT, permeabilized with 0.3% Triton X-100 in PBS for 10 min and blocked in 10% normal goat serum (Millipore Sigma, St. Louis, MO, USA) for 1 h at RT. The Nrf2-568 antibody was incubated overnight at 4 °C in a humidified chamber protected from light. The slides were washed 3 times in PBS and Fluoroshield (Millipore Sigma) containing DAPI was added to stain nuclei and mount coverslips onto slides. Immunofluorescent staining was imaged using a 710 Confocal laser scanning microscope (Zeiss, White Plains, NY, USA) and nuclear localization of Nrf2 was assessed using Image J software using a previously described protocol [71].

### 4.9. Measurement of Intracellular ROS and Mitochondrial Superoxide

ROS production in the cell lines was determined using fluorescent dye chloromethyl-2′,7′-dichlorofluorescein diacetate (H2DCFDA, Thermo Fisher Scientific, Waltham, MA, USA) and mitochondrial superoxide production was determined using fluorescent compound MitoSOX™ Red (Thermo Fisher Scientific, Waltham, MA, USA). Adherent cells were plated at 4000 cells/well in a 96 well clear bottom black plate overnight before treatment. Cells grown in TIC-enriching conditions were grown in ULA plates for 3 days in TEM, treated with the drugs and then transferred to a 96 well clear-bottom black plate. Cells were treated with drugs for 6 h at 37 °C, were washed with PBS and incubated with 10 μM H2DCFDA at 37 °C for 30 min in PBS to measure ROS, or 5μM MitoSOX at 37 °C for 10 min to measure mitochondrial superoxide, protected from light to load the fluorescent dye. After loading cells were washed twice with PBS and resuspended in 100 μL PBS. Florescence was measured on a SpectraMax iD3 (Molecular Devices) microplate reader.

### 4.10. In Vivo Studies

All animal studies were approved by the NCI Animal Care and Use Committee, IACUC Number MOB-025-1. Intra-bursal xenografts were generated by injection of 1 × 10^6^ OVCAR8 cells in 5 μL PBS into the right ovarian bursa of 8-week-old female athymic Nu/Nu mice. For controls, 5 μL PBS was injected into the left ovarian bursa of each mouse. After 14 days, ovaries were removed, and mice were allowed to recover for 14 days before receiving citrate buffer for vehicle control or carboplatin (50 mg/kg) and paclitaxel (27 mg/kg) intraperitoneally once per week for each drug for 3 weeks. Following these treatments, salinomycin (5 mg/kg) or disulfiram (10 mg/kg) or citrate buffer for vehicle controls was administered intraperitoneally, three times per week for 3 weeks. The animals were monitored for health and survival in days was recorded as mice met NIH Animal Care and Use Committee-approved humane criteria for euthanasia.

### 4.11. Statistical Analysis

In vitro assays were performed in triplicate on three independent occasions and were analyzed with *t*-tests or one-way ANOVA where applicable. Results are presented as mean ± SEM for in vitro data, with *p* values ≤ 0.05 considered significant. Kaplan-Meier analysis was used to analyze overall survival for in vivo studies, and Mantel-Cox log-rank was used to compare groups. Statistical analyses were performed using Prism 7.0 software (GraphPad, San Diego, CA, USA).

## 5. Conclusions

There is a great need for therapeutic options for maintenance therapy in relapsed ovarian cancer, particularly for BRCA wild-type patients and patients that do not respond to PARP inhibitor therapy. In this study we used cell lines representing chemoresistant, HR proficient OC in a comprehensive drug screen and in RNA sequencing analysis to identify drugs and pathways that were enriched in the TIC population. We investigated the drugs disulfiram, bardoxolone methyl, elesclomol and salinomycin for activity in targeting the TIC population that promotes disease recurrence in vitro and in vivo. We found that TICs were more sensitive to oxidative stress and the drugs disulfiram and salinomycin were effective in preventing relapse after surgical resection and carboplatin-paclitaxel chemotherapy in an in vivo relapse model. These findings indicate the therapeutic potential of the development of drugs that enhance oxidative stress and inhibit ALDH activity as maintenance therapy to prevent relapse in OC.

## Figures and Tables

**Table 1:** Summary table of half maximal toxicity nM concentrations of the drugs against each cell line under adherent or TIC culture conditions.

	OV90		OVCAR8		CAOV3	
Drug	Adherent	Spheroid	Adherent	Spheroid	Adherent	Spheroid
**Disulfiram**	213.68	228.88	> 2000 ^1^	514.62	> 2000 ^1^	192.69
**Bardoxolone methyl**	135.09	105.05	399.54	418.07	150.39	100.16
**Elesclomol**	28.92	59.89	160.49	30.86	> 500 ^1^	145.89
**Salinomycin**	500.20	419.55	720.25	294.88	1495.02	2339.74

^1^ Where the half maximal toxicity was not reached at the highest concentration, it is represented as greater than (>) the highest dose.

## Supplementary Materials

The following are available online at https://www.mdpi.com/2072-6694/12/6/1645/s1, Figure S1: Oxidative stress in OVCAR8 cells grown adherently. Figure S2: Optimization of number of chemotherapy cycles for modelling in vivo relapse, Table S1: The relative in vitro LD_50_ for each of the MIPE 5.0 compounds for OV90 grown under adherent and TIC-enriching conditions.
