# Supplementary material for: Drugs Targeting Tumor-Initiating Cells Prolong Survival in a Post-Surgery, Post-Chemotherapy Ovarian Cancer Relapse Model
Source: Cancers (Basel). 2020 Jun 21;12(6):1645. doi: 10.3390/cancers12061645 (PMC7352549; doi:10.3390/cancers12061645)
Supplement: Supplementary file 1 [file cancers-12-01645-s001.zip › cancers-823450-supplementary/cancers-823450 Supplementary layout_BHedit.pdf]

cancers

MDPI

Supplementary Materials

Drugs Targeting Tumor-Initiating Cells Prolong Survival in a Post-Surgery, Post-Chemotherapy Ovarian Cancer Relapse Model

Brittney S. Harrington, Michelle K. Ozaki, Michael W. Caminear, Lidia F. Hernandez, Elizabeth Jordan, Nicholas J. Kalinowski, Ian S. Goldlust, Rajarshi Guha, Marc Ferrer, Craig Thomas, Jyoti Shetty, Bao Tran, Nathan Wong, Carrie D. House and Christina M. Annunziata

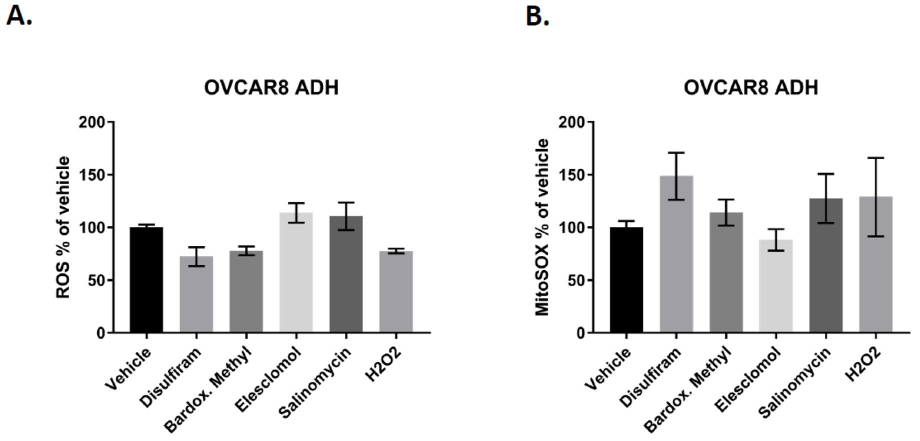

Figure S1. Oxidative stress in OVCAR8 cells grown adherently. (**A**) Intracellular ROS measured after 6H of treatment with indicated drugs, expressed as a percentage of vehicle control. (**B**) Mitochondrial Superoxide (MitoSOX) measured after 6H of treatment with indicated drugs, expressed as a percentage of vehicle control.

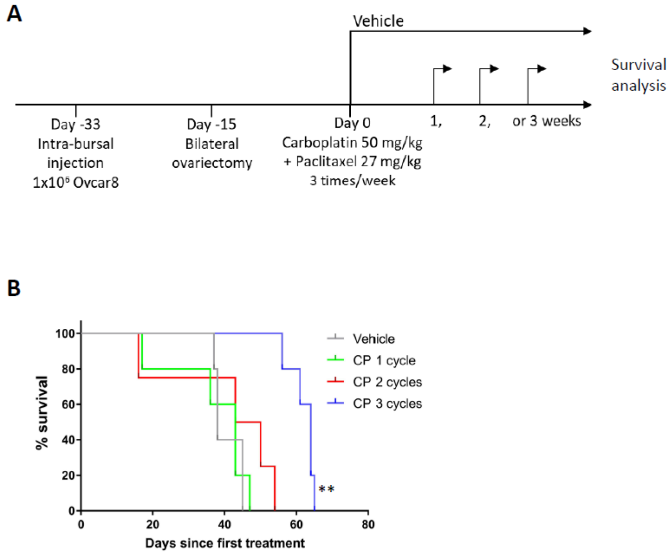

Figure S2. Optimization of number of chemotherapy cycles for modelling *in vivo* relapse. (**A**) Schematic of the *in vivo* relapse model. (**B**) Kaplan-Meier survival analysis of mice treated with vehicle only (grey), 1 cycle (green), 2 cycles (red) or 3 cycles (blue) of Carboplatin and Paclitaxel. Survival was measured in days since the first treatment $^{**}p < 0.01$.

© 2020 by the authors. Licensee MDPI, Basel, Switzerland. This article is an open access article distributed under the terms and conditions of the Creative Commons Attribution (CCBY) license (http://creativecommons.org/licenses/by/4.0/).
